# Alopécies du scalp d’étiologies atypiques

**DOI:** 10.11604/pamj.2018.29.213.14533

**Published:** 2018-04-13

**Authors:** Mouna Ejjiyar, Moulay Driss El Amrani

**Affiliations:** 1 Service de Chirurgie Plastique, CHU Mohammed VI, Marrakech, Maroc

**Keywords:** Alopécie, scalp, étiologies atypiques, Alopecia, scalp, unusual cause

## Abstract

Alopecia is defined as a congenital or temporary absence of hair or even as total or partial hair loss. Clinical manifestation is more marked on the scalp and it is more common in men than in women. Nevertheless, a set of extrinsic factors may be responsible for scarring alopecia of the scalp, including tumors, infections or even loss of substance secondary to thermal burns. On the basis of these illustrations, we here report three cases of patients with scarring alopecia of the scalp secondary to so-called “atypical” causes. The first case (A) is that of a 6-year-old child presenting with alopecia on the right parietal scalp. In-depth interview with his mother allowed to discover the cause of the loss of substance: cutaneous necrosis following laborious delivery by vacuum extraction. The second illustration (B) shows the case of a 40-year-old woman seen in consultation for cutaneous necrosis of the occipital scalp secondary to chemical burn. The patient reported the application of a chemical product in order to fix hairstyle. Finally, the third case (C) is that of a 27-year old patient followed-up for bilateral frontoparietal alopecia secondary to the application of poisonous plant having, according to her beliefs, nourishing virtues for the hair.

## Image en médecine

L'alopécie est définie par l'absence congénitale ou temporaire, voire par la chute totale ou partielle, du cheveu. Cette manifestation est plus marquée au niveau du cuir chevelu, et reste plus fréquente chez l'homme que chez la femme. Néanmoins, on distingue un ensemble de facteurs extrinsèques pouvant être responsable d'une alopécie cicatricielle au niveau du scalp, notamment l'origine tumorale, infectieuse, voire même les pertes de substance secondaires à des brûlures thermiques. Sur ces illustrations, nous présentons trois cas de patients présentant des alopécies cicatricielles du scalp secondaires à des étiologies dites « atypiques ». Le premier cas (A) est celui d'un enfant âgé de 6 ans ayant consulté pour une alopécie pariétale droite. L'interrogatoire approfondi avec la maman a permis de retrouver l'étiologie de la perte de substance: une nécrose cutanée suite à un accouchement laborieux avec extraction du bébé par ventouse. La deuxième illustration (B) montre le cas d'une patiente âgée de 40 ans, vue en consultation pour une nécrose cutanée au niveau du scalp occipital, secondaire à une brûlure chimique. En effet, la patiente avait rapporté la notion d'application d'un produit chimique dans le but de fixer sa coiffure. Enfin, le troisième cas (C), est celui d'une jeune patiente de 27 ans, suivie pour prise en charge d'une alopécie fronto-pariétale bilatérale secondaire à l'application d'une plante toxique, ayant selon ses croyances, des vertues nourrissantes pour le cheveu.

**Figure 1:**
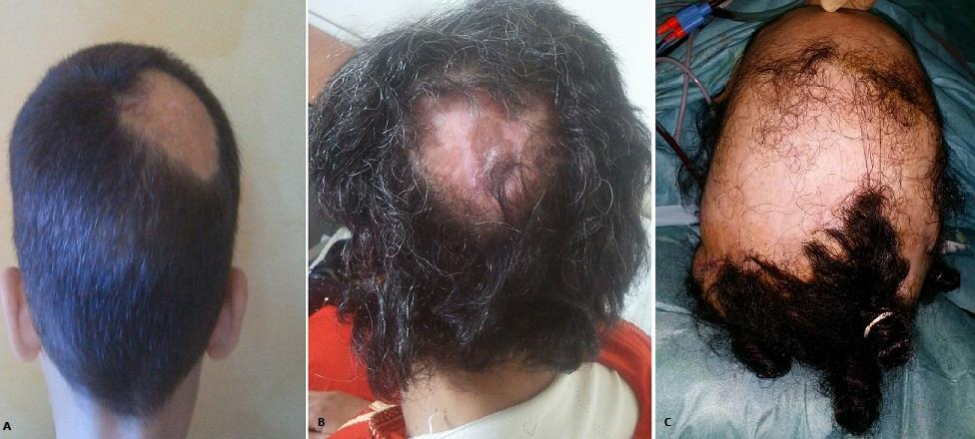
(A,B,C) alopecies du scalp d’étiologies atypiques

